# Supplementary figures and images for: Parallel altitudinal clines reveal trends in adaptive evolution of genome size in Zea mays
Source: PLoS Genet. 2018 May 10;14(5):e1007162. doi: 10.1371/journal.pgen.1007162 (PMC5944917; doi:10.1371/journal.pgen.1007162)

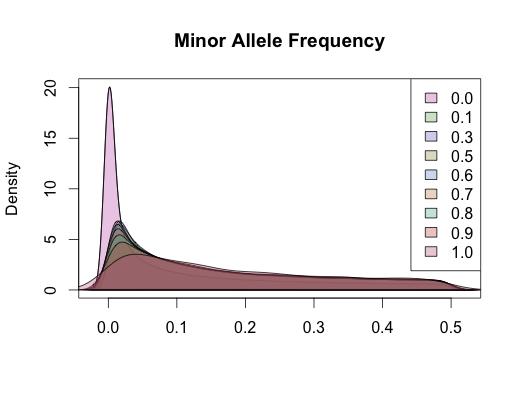

Supplement: S1 Fig — Key indicates percent of site coverage, ranging from unfiltered (0.0) to a requirement of full data presence across individuals (1.0). The spectrum begins to shift after a 60% site coverage requirement. (TIFF) [file pgen.1007162.s001.tiff]

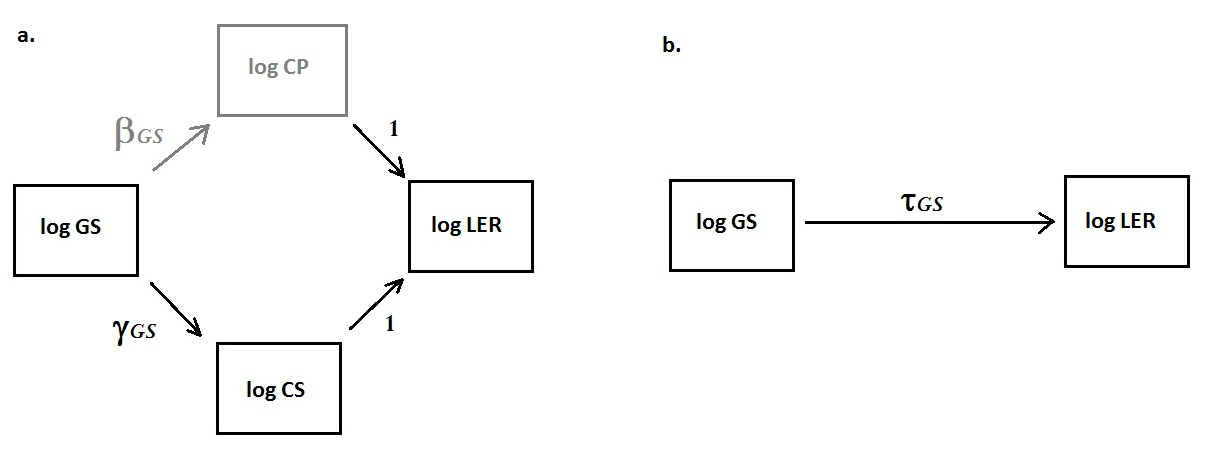

Supplement: S2 Fig — Arrows indicate predictor-outcome relationships and are annotated with model coefficients (slopes) from equations 9-11. (A) Genome Size (GS) predicts Leaf Elongation Rate (LER) through the mediators Cell Size (CS) and Cell Production rate (CP). CP, shown in grey, is not directly observed. The unit coefficients connecting log LER with log CP and log CS reflect the assumption LER = CS * CP (Eq 3). (B) Marginal model for the effect of GS on LER (equation 10). (TIFF) [file pgen.1007162.s002.tiff]

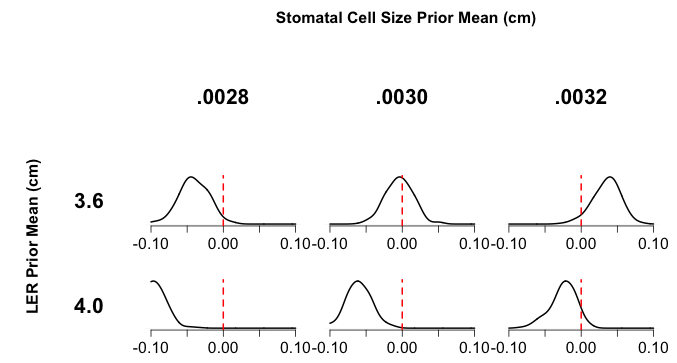

Supplement: S3 Fig — (TIFF) [file pgen.1007162.s003.tiff]

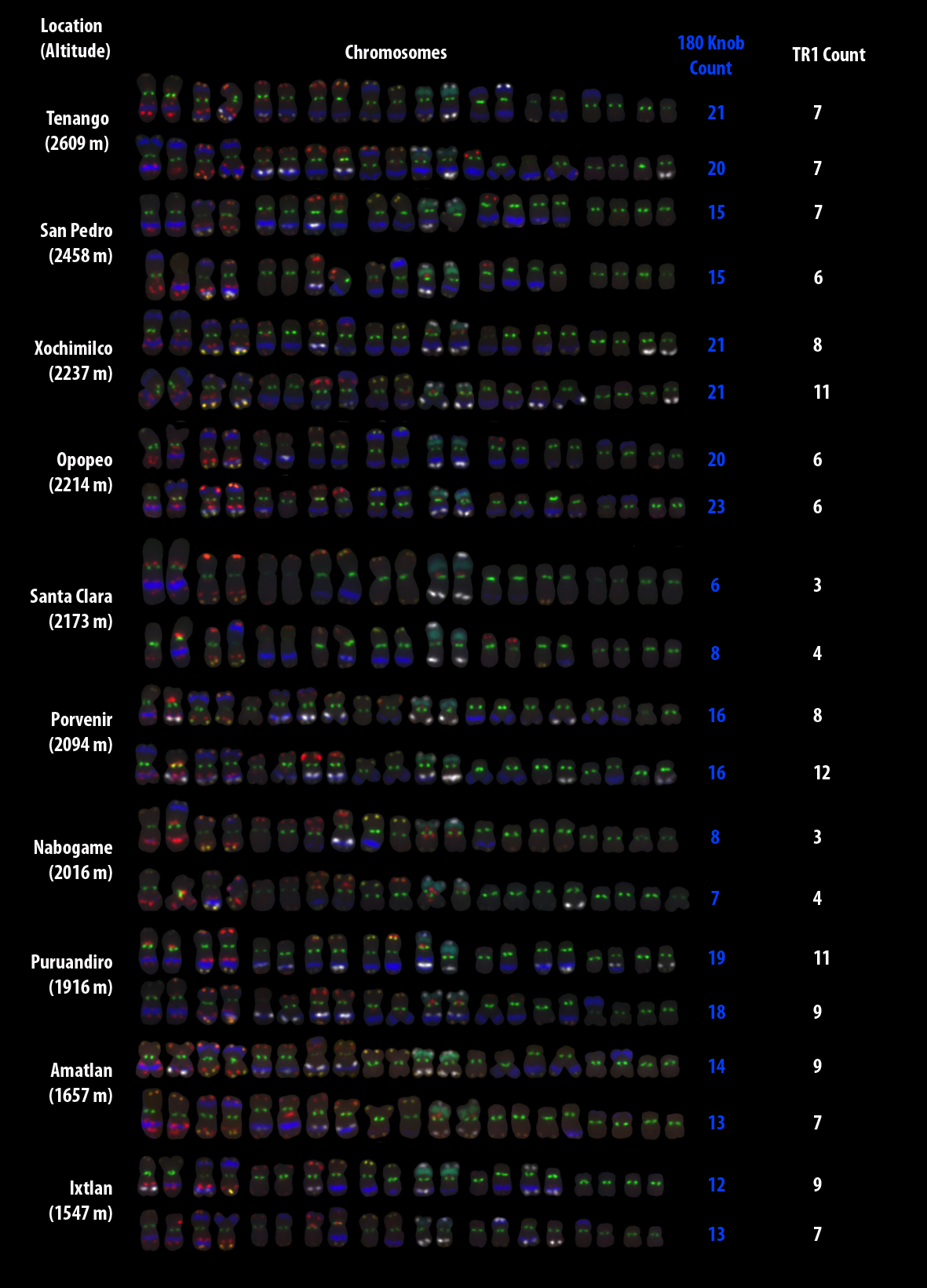

Supplement: S4 Fig — Counts of cytological 180bp knobs (blue) and TR1 knobs (white) are shown to the right of each individual. Other stained repeats are CentC and subtelomere 4-12-1 (green), 5S ribosomal gene (yellow), Cent4 (orange), NOR (blue-green), and TAG microsatellite 1-26-2 and subtelomere 1.1 (red). For further staining information, see [40]. (TIFF) [file pgen.1007162.s004.tiff]

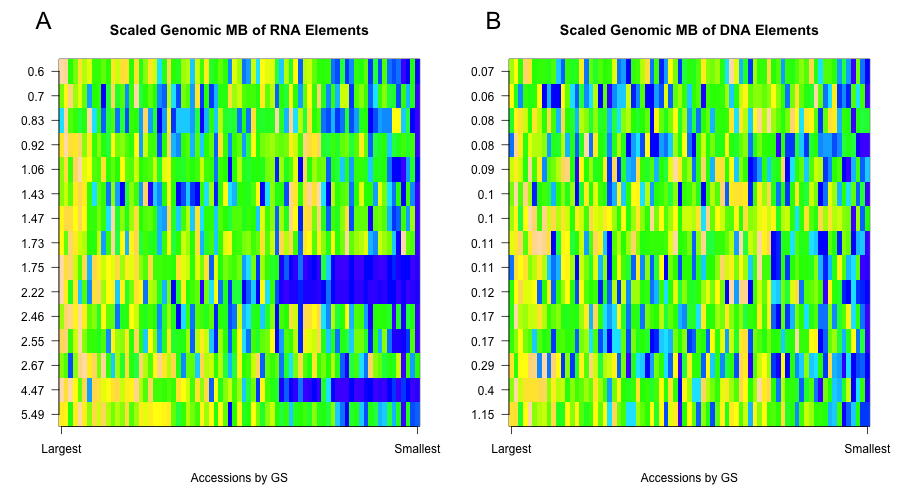

Supplement: S5 Fig — The y-axis indicates the average abundance in Mb of a given TE subfamily. The fifteen highest abundance subfamilies are shown. The x-axis are maize landraces accessions ordered by genome size, with the largest genome size accessions on the left. Values plotted are bp measures scaled from 0 (blue) to 1 (yellow) per row. (TIFF) [file pgen.1007162.s005.tiff]

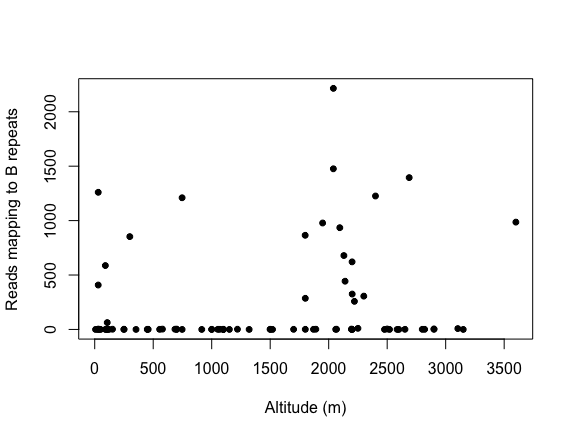

Supplement: S6 Fig — (TIFF) [file pgen.1007162.s006.tiff]

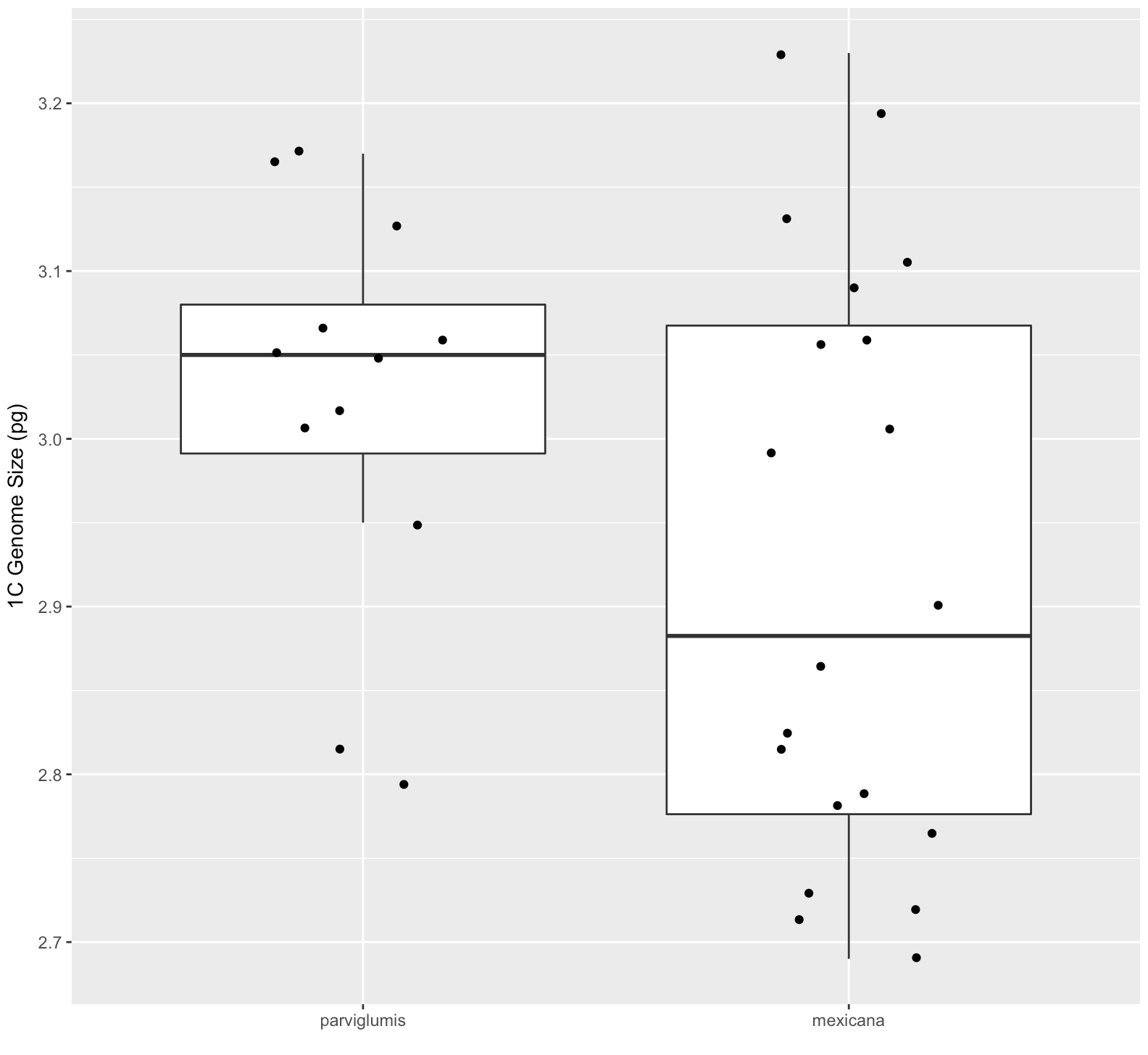

Supplement: S7 Fig — Points indicating individual genome size estimates are jittered around the center. With a parametric t-test of unequal variance, the one sided p-value is 0.03. Using a non-parametric Wilcoxon test, the one tailed p-value is 0.06. (TIFF) [file pgen.1007162.s007.tiff]

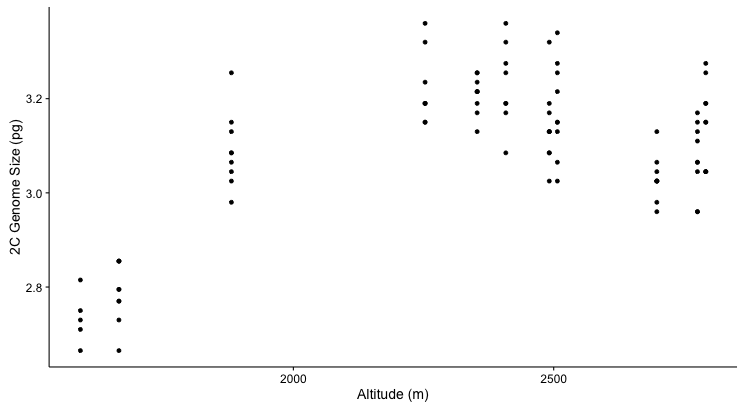

Supplement: S8 Fig — All samples, including low altitude populations, are shown. (TIFF) [file pgen.1007162.s008.tiff]

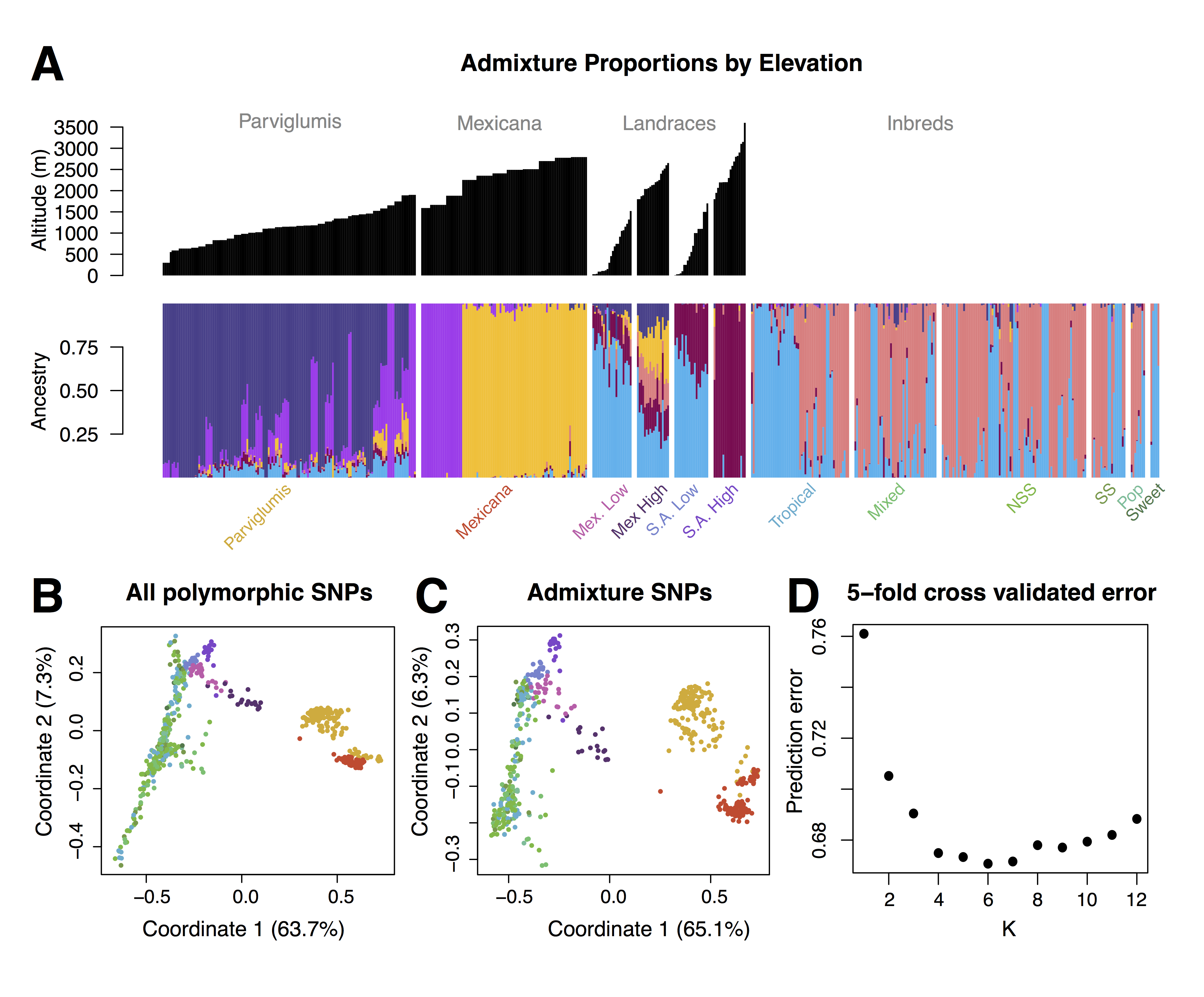

Supplement: S9 Fig — (A) Admixture plots for K = 6, with altitude of accessions shown above. Mexicana populations and maize landraces are those used in this study. We include parviglumis [77] and maize inbreds [76]. (B) and (C) Multi-dimensional scaling analyses showing clustering of whole genome SNPs and those used to generate the admixture plot. Points are color coded based on the label underneath the admixture plot. (D) 5-fold cross validated error as estimated by Admixture, indicating the best estimate of number of populations, K. (TIFF) [file pgen.1007162.s009.tiff]
