# Supplementary material for: Parallel altitudinal clines reveal trends in adaptive evolution of genome size in Zea mays
Source: PLoS Genet. 2018 May 10;14(5):e1007162. doi: 10.1371/journal.pgen.1007162 (PMC5944917; doi:10.1371/journal.pgen.1007162)
Supplement: S1 Appendix — (PDF) [file pgen.1007162.s010.pdf]

S1 Appendix. Path Model

As shown below, a path model enables the relationship between cell production rate and genome size to be inferred by fitting regressions for log CS on log GS, and log LER on log GS. The general approach is described as a model with two “mediators” in Mackinnon [84]. We took a computational Bayesian approach to fitting the path model after early experiments with likelihood-based methods indicated numerical instabilities.

In detail, the first of the regression models is, for cell c of seedling s:

$$log(CS)_{s,c} = M_{CS,s} + Z_{CS,s} + \gamma_{GS} * log(GS_s) + E_{CS,s,c} \quad (6)$$

where $M_{CS,s}$ is a random intercept for the maternal parent of seedling s, $Z_{CS,s}$ is a random intercept for seedling s, and $E_{CS,s,c}$ is a cell-level error term. Inclusion of an overall mean, μ, in the regression equation along with the random intercepts led to numerical instabilities apparently related to poor model identification. In effect, the random intercepts– when properly parameterized– take the place of an overall mean. We found that informative priors for $M_{CS,s}$ and $Z_{CS,s}$ were necessary: for the final model we used Gaussian priors with mean log(0.003) and standard deviations $\sigma_{M_{CS}}$ and $\sigma_{Z_{CS}}$, respectively. This prior mean is the natural logarithm of a typical stomatal cell size, 0.003cm. We expect one of the two random intercepts to assume a greater role in capturing the overall mean. Centering both priors at log(0.003) reflects our indifference to the outcome of this contest. The prior for $E_{CS,s,c}$ is Gaussian with mean zero and standard deviation $\sigma_{E_{CS}}$. The coefficient γ_{GS} has a Gaussian prior with mean zero and standard deviation 5.0. We used half-Cauchy priors for the standard deviations $\sigma_{E_{CS}}$, $\sigma_{M_{CS}}$ and $\sigma_{Z_{CS}}$.

The second of two regression models– for log LER on log GS– is derived from a model reflecting primary observations of leaf *length* (LL) on successive days of seedling growth. The observation-level model for seedling s at time t is:

$$log(LL)_{s,t} = M_{LL,s} + Z_{LL,s} + \tau_{GS} * log(GS_s) + (M_{LER,s} + Z_{LER,s}) * log\ t + E_{LL,s,t} \quad (7)$$

where $M_{LL,s}$ and $Z_{LL,s}$ are respectively maternal and seedling random intercepts, $M_{LER,s}$ and $Z_{LER,s}$ are maternal and seedling random slopes and $E_{LL,s,t}$ is an error term. The random slopes $M_{LER,s}$ and $Z_{LER,s}$ allow for idiosyncratic growth rates. Natural logarithms on the right- and left-hand sides imply power-law relationships between leaf length, time and genome size in their original units of measurement. As in model 6, informative priors were necessary for model identification. The final model has Gaussian priors with mean log(4.8) and standard deviations $\sigma_{M_{LL}}$ and $\sigma_{Z_{LL}}$ for $M_{LL,s}$ and $Z_{LL,s}$, respectively. This prior mean is the natural logarithm of a typical leaf elongation rate, 4.8cm– the increment of leaf length that could be expected after a day's growth ($t = 1$). The priors for $M_{LER,s}$ and $Z_{LER,s}$ are Gamma with shape and rate both equal to 1.0 (*i.e.* with mean 1.0, reflecting linear growth). As for model 6, the prior for $E_{LL,s,t}$ is Gaussian with mean zero and standard deviation $\sigma_{E_{LL}}$, the coefficient τ_{GS} has a Gaussian prior with mean zero and standard deviation 5.0, and $\sigma_{M_{LL}}$, $\sigma_{Z_{LL}}$ and $\sigma_{E_{LL}}$ have half-Cauchy priors.

The model for leaf *elongation rate* is subsequently obtained by differentiation of $LL_{s,t}$ with respect to time:

$$\begin{aligned} log(LER)_{s,t} = log(\frac{d}{dt}LL_{s,t}) &= M_{LL,s} + Z_{LL,s} + \tau_{GS} * log(GS_s) \\ &+ (M_{LER,s} + Z_{LER,s} - 1) * log\ t + log(M_{LER,s} + Z_{LER,s}) + E_{LL,s,t}. \quad (8) \end{aligned}$$

Equations 6 and 8 are placed in context by use of the path diagrams in S2 Fig. Equation 6 is the sub-model connecting log CS to log GS in the left-hand diagram, while equation 8 is a marginal model connecting log LER to log GS, illustrated by the

right-hand diagram. The two path diagrams imply two expressions for the same quantity, log LER, which can be equated to produce an estimate of β_{GS}. The derivation is simplified by taking expected values of the random effects in 6 and 8, and fixing the time horizon at a single day, though the numerical estimates we report come from 6 and 8 in full detail, as displayed above. Subsequently we define

$$\mathbb{E}[M_{CS,s} + Z_{CS,s} + E_{CS,s,c}] = \gamma_0,$$

$$\mathbb{E}[M_{LL,s} + Z_{LL,s} + log(M_{LER,s} + Z_{LER,s}) + E_{LL,s,t}] = \tau_0,$$

and set $t = 1$ in equation 8. Equations 6 and 8 then simplify as

$$log(CS) = \gamma_0 + \gamma_{GS} * log(GS) \tag{9}$$

$$log(LER) = \tau_0 + \tau_{GS} * log(GS) \tag{10}$$

respectively. These are joined by a similar equation for the unobserved variable:

$$log(CP) = \beta_0 + \beta_{GS} * log(GS). \tag{11}$$

Working from the relationship $LER = CS * CP$, or alternatively from log LER backward to its precedents in the left-hand path diagram, we find:

$$\begin{aligned} log(LER) &= log(CS) + log(CP) && (12) \\ &= \gamma_0 + \gamma_{GS} * log(GS) + \beta_0 + \beta_{GS} * log(GS), && (13) \end{aligned}$$

substituting right-hand sides of 9 and 11. Equating expressions 13 and 10 and collecting terms, the coefficient β_{GS} is recovered as $\beta_{GS} = \tau_{GS} - \gamma_{GS}$.
