# Supplementary material for: Parallel altitudinal clines reveal trends in adaptive evolution of genome size in Zea mays
Source: PLoS Genet. 2018 May 10;14(5):e1007162. doi: 10.1371/journal.pgen.1007162 (PMC5944917; doi:10.1371/journal.pgen.1007162)
Supplement: S1 Table — (PDF) [file pgen.1007162.s011.pdf]

**S1 Table. Description of data sets used in each analysis.**

| Data Group | Genome Size | WGS sequencing Individual | Genotyping Individual | FISH performed? | Used in |
|---|---|---|---|---|---|
| 77 Landraces | Per Individual | Same individual as GS | Different individual than GS but within accession | No | Selection study |
| 93 Mexicana individuals from 11 Populations | Per Individual | Same individual as GS | Same individual as GS | No | Selection study |
| 6 Parviglumis Populations | 2 Individuals per population | NA | NA | No | Teosinte pilot study |
| 10 Mexicana populations | 2 Individuals per population | 9-12 Individuals per population | NA | Yes, different individuals than GS | Teosinte pilot study |
