# Supplementary material for: Parallel altitudinal clines reveal trends in adaptive evolution of genome size in Zea mays
Source: PLoS Genet. 2018 May 10;14(5):e1007162. doi: 10.1371/journal.pgen.1007162 (PMC5944917; doi:10.1371/journal.pgen.1007162)
Supplement: S2 Table — (PDF) [file pgen.1007162.s012.pdf]

S2 Table. Geographic information for maize landrace accessions.

Accession ID	Region	Altitude (m)	Latitude	Longitude
RIMMA0388.1	SAL	1500	6.85	-75.28333333
RIMMA0389.1	SAL	7	10.38333333	-74.88333333
RIMMA0390.1	SAL	353	4.516666667	-75.63333333
RIMMA0392.1	SAL	555	1.75	-75.58333333
RIMMA0393.1	SAL	100	8.316666667	-75.15
RIMMA0394.2	SAL	1000	4.783333333	-74.68333333
RIMMA0395.2	SAL	30	8.5	-77.26666667
RIMMA0396.1	SAL	1100	2.583333333	-75.3
RIMMA0397.1	SAL	50	11.55	-72.91666667
RIMMA0398.1	SAL	27	9.433333333	-75.7
RIMMA0399.1	SAL	250	10.18333333	-74.05
RIMMA0403.2	SAL	1000	1.25	-77.51666667
RIMMA0404.1	SAL	1500	7.3	-72.51666667
RIMMA0406.1	SAL	450	4.966666667	-74.9
RIMMA0409.1	ML	107	15.43333333	-92.9
RIMMA0416.1	MH	2140	29.35	-107.75
RIMMA0417.1	MH	2040	28.55	-107.4833333
RIMMA0418.1	MH	2040	28.56666667	-107.4833333
RIMMA0421.1	MH	2250	19.85	-97.98333333
RIMMA0422.1	MH	2200	19.1	-98.3
RIMMA0423.1	MH	2095	29.21666667	-108.1333333
RIMMA0424.1	MH	2400	27.98333333	-107.5833333
RIMMA0425.1	MH	2130	26.81666667	-107.0666667
RIMMA0426.1	SAH	2500	-9.066666667	-77.81666667
RIMMA0428.1	SAL	700	-9.3	-76
RIMMA0430.1	SAH	2585	-9.166666667	-77.73333333
RIMMA0431.1	SAH	2900	-8.65	-77.08333333
RIMMA0433.1	ML	457	14.71666667	-89.5
RIMMA0436.1	SAH	2200	-6.15	-77.91666667
RIMMA0437.1	SAH	2688	-9.266666667	-77.63333333
RIMMA0438.1	SAH	2820	-8.7	-77.38333333
RIMMA0441.1	ML	1320	19.16666667	-96.96666667
RIMMA0462.1	SAL	1700	1.6	-77.15
RIMMA0464.1	SAH	1800	-12.33333333	-74.7
RIMMA0465.1	SAH	2300	-5.566666667	-79.53333333
RIMMA0466.1	SAH	3600	-14.31666667	-72.91666667
RIMMA0467.1	SAH	2800	-13.58333333	-72.91666667
RIMMA0468.1	SAH	3150	-9.383333333	-77.16666667
RIMMA0473.1	SAH	3104	1.083333333	-77.61666667
RIMMA0614.1	MH	2060	19.95	-103.7666667
RIMMA0615.1	ML	152	20.13333333	-97.2
RIMMA0616.1	MH	1800	20.81666667	-102.7666667
RIMMA0619.1	ML	747	18.35	-99.53333333
RIMMA0620.1	MH	1799	20.21666667	-100.8833333
RIMMA0621.1	MH	1870	21.11666667	-101.6833333
RIMMA0623.1	MH	2520	20.03333333	-103.6833333
RIMMA0625.1	MH	2600	19	-97.38333333
RIMMA0628.1	ML	300	23.31666667	-99.01666667
RIMMA0630.1	MH	2220	19.8	-97.25

RIMMA0657.1	SAH	2201	-17.5	-65.66666667
RIMMA0658.1	SAH	1948	-21.83333333	-64.13333333
RIMMA0661.1	SAH	2195	-2.85	-78.66666667
RIMMA0662.1	SAH	2195	0	-78
RIMMA0663.1	SAH	2067	0.433333333	-78.2
RIMMA0667.1	SAH	2201	-21.83333333	-64.13333333
RIMMA0671.1	MH	2477	14.76666667	-91.25
RIMMA0674.1	MH	2652	19.28333333	-99.66666667
RIMMA0680.1	MH	1890	20.36666667	-102.1666667
RIMMA0690.1	SAL	250	8.316666667	-73.63333333
RIMMA0691.1	SAL	1098	6.55	-73.13333333
RIMMA0696.1	ML	30	16.51666667	-90.16666667
RIMMA0700.1	ML	579	16.75	-93.16666667
RIMMA0701.1	ML	686	16.6	-92.71666667
RIMMA0702.1	ML	1052	14.46666667	-90.75
RIMMA0703.1	ML	30	20.83333333	-88.51666667
RIMMA0708.1	SAL	1098	-3.5	-78.6
RIMMA0709.1	ML	747	16.5	-92.5
RIMMA0710.1	ML	91	15.33333333	-92.63333333
RIMMA0712.1	ML	1220	15.28333333	-90.25
RIMMA0716.1	ML	91	15.31666667	-92.66666667
RIMMA0720.1	ML	39	15.46666667	-88.85
RIMMA0721.1	ML	915	14.61666667	-90.08333333
RIMMA0727.1	ML	1151	14.4	-90.46666667
RIMMA0729.1	ML	122	15.4	-89.66666667
RIMMA0730.1	ML	1067	14.48333333	-90.8
RIMMA0731.1	ML	1520	16.78333333	-96.66666667
RIMMA0733.1	ML	107	16.56666667	-94.61666667
