# Supplementary material for: Parallel altitudinal clines reveal trends in adaptive evolution of genome size in Zea mays
Source: PLoS Genet. 2018 May 10;14(5):e1007162. doi: 10.1371/journal.pgen.1007162 (PMC5944917; doi:10.1371/journal.pgen.1007162)
Supplement: S3 Table — (PDF) [file pgen.1007162.s013.pdf]

S3 Table. Measures of genome size from two individuals from each of the 10 populations used in FISH to sequence correlation (Fig. 2).

Accession	Subspecies	Ind1	Ind2	Altitude(m)
RIMME0021	mexicana	6.12	6.01	2094
RIMME0026	mexicana	6.21	6.11	2214
RIMME0028	mexicana	5.65	5.53	1916
RIMME0029	mexicana	5.46	5.58	1547
RIMME0030	mexicana	5.98	5.8	2458
RIMME0031	mexicana	6.26	6.39	2609
RIMME0032	mexicana	5.63	5.73	2016
RIMME0033	mexicana	5.43	5.44	1657
RIMME0034	mexicana	5.56	5.38	2173
RIMME0035	mexicana	6.18	6.46	2237
RIMPA0071	parviglumis	6.1	6.1	985
RIMPA0086	parviglumis	6.01	5.9	982
RIMPA0087	parviglumis	5.59	5.63	590
RIMPA0096	parviglumis	6.12	6.03	1528
RIMPA0135	parviglumis	6.25	6.34	880
RIMPA0142	parviglumis	6.33	6.13	1103
