# Supplementary material for: Parallel altitudinal clines reveal trends in adaptive evolution of genome size in Zea mays
Source: PLoS Genet. 2018 May 10;14(5):e1007162. doi: 10.1371/journal.pgen.1007162 (PMC5944917; doi:10.1371/journal.pgen.1007162)
Supplement: S4 Table — (PDF) [file pgen.1007162.s014.pdf]

S4 Table. Geographic information for teosinte populations used in selection studies.

Population	Latitude	Longitutde	Altitude (M)	Locality
TZ	18.975744992	-99.069713429	1665.116699	Tepoztlán
FP	19.211739153	-99.126956714	2506.984619	S Francisco Pedregal
MT	19.211638905	-98.808725439	2352.93457	S Mateo Tezoquipan
DA	19.145727055	-98.862849986	2408.450439	Tenango del Aire
MC	19.076366471	-98.84329861	2501.216797	S Matías Cuijingo
M	18.953779545	-99.501451766	1882.853516	Malinalco
TC	19.260426778	-99.722122969	2776.151855	Toluca
TX	19.504571417	-98.922480522	2252.718018	Texcoco de Mora
CL	19.151357012	-99.616249725	2697.564941	Calimaya Lower
CU	19.160612	-99.632908	2792	Calimaya Upper
AM	18.97155	0-99.036917	1591	Amatlán
