# Supplementary material for: Parallel altitudinal clines reveal trends in adaptive evolution of genome size in Zea mays
Source: PLoS Genet. 2018 May 10;14(5):e1007162. doi: 10.1371/journal.pgen.1007162 (PMC5944917; doi:10.1371/journal.pgen.1007162)
Supplement: S5 Table — (PDF) [file pgen.1007162.s015.pdf]

**S5 Table. Genome size estimates and altitudinal information for mexicana populations**

| Population | Accession | DNA (pg/2C) | Altitude (m) |
|---|---|---|---|
| Tz | 13 | 5.59 | 1665 |
| Tz | 2 | 5.46 | 1665 |
| Tz | 3 | 5.33 | 1665 |
| Tz | 4 | 5.71 | 1665 |
| Tz | 19 | 5.71 | 1665 |
| Tz | 23 | 5.54 | 1665 |
| Tz | 9 | 5.59 | 1665 |
| Tz | 10 | 5.54 | 1665 |
| Tz | 12 | 5.71 | 1665 |
| Fp | 1 | 6.51 | 2507 |
| Fp | 2 | 6.26 | 2507 |
| Fp | 3 | 6.3 | 2507 |
| Fp | 4 | 6.43 | 2507 |
| Fp | 5 | 6.3 | 2507 |
| Fp | E | 6.05 | 2507 |
| Fp | 8 | 6.68 | 2507 |
| Fp | 9 | 6.13 | 2507 |
| Fp | 12 | 6.55 | 2507 |
| Mt | 1 | 6.47 | 2353 |
| Mt | 4 | 6.34 | 2353 |
| Mt | 5 | 6.51 | 2353 |
| Mt | 7 | 6.26 | 2353 |
| Mt | 8 | 6.38 | 2353 |
| Mt | 9 | 6.43 | 2353 |
| Mt | 10 | 6.51 | 2353 |
| Mt | 11 | 6.43 | 2353 |
| Mt | 12 | 6.43 | 2353 |
| Da | 1 | 6.72 | 2408 |
| Da | 2 | 6.64 | 2408 |
| Da | 3 | 6.38 | 2408 |
| Da | 5 | 6.51 | 2408 |
| Da | 6 | 6.47 | 2408 |
| Da | 8 | 6.17 | 2408 |
| Da | 9 | 6.55 | 2408 |
| Da | 10 | 6.34 | 2408 |
| Da | 11 | 6.38 | 2408 |
| Mc | 1 | 6.34 | 2491.5 |
| Mc | 2 | 6.05 | 2491.5 |
| Mc | 3 | 6.26 | 2491.5 |
| Mc | 5 | 6.26 | 2491.5 |
| Mc | 6 | 6.38 | 2491.5 |
| Mc | 9 | 6.17 | 2491.5 |
| Mc | 10 | 6.17 | 2491.5 |
| Mc | 11 | 6.26 | 2491.5 |
| Mc | 12 | 6.64 | 2491.5 |
| M | 2 | 6.26 | 1881 |
| M | 4 | 5.96 | 1881 |
| M | 5 | 6.05 | 1881 |

| | | | |
|---|---|---|---|
| M | 6 | 6.13 | 1881 |
| M | 7 | 6.17 | 1881 |
| M | 8 | 6.09 | 1881 |
| M | 9 | 6.17 | 1881 |
| M | 10 | 6.51 | 1881 |
| M | 11 | 6.3 | 1881 |
| Tc | 1 | 6.09 | 2776 |
| Tc | 2 | 6.13 | 2776 |
| Tc | 3 | 6.22 | 2776 |
| Tc | 4 | 5.92 | 2776 |
| Tc | 5 | 6.34 | 2776 |
| Tc | E/1 | 5.92 | 2776 |
| Tc | 7 | 6.13 | 2776 |
| Tc | 8 | 6.26 | 2776 |
| Tc | 12 | 6.3 | 2776 |
| Tx | 2 | 6.64 | 2253 |
| Tx | 3 | 6.47 | 2253 |
| Tx | 4 | 6.38 | 2253 |
| Tx | 7 | 6.38 | 2253 |
| Tx | 8 | 6.38 | 2253 |
| Tx | 9 | 6.3 | 2253 |
| Tx | 10 | 6.3 | 2253 |
| Tx | 11 | 6.72 | 2253 |
| Tx | 12 | 6.72 | 2253 |
| Cl | 1 | 5.96 | 2698 |
| Cl | 2 | 6.13 | 2698 |
| Cl | 4 | 6.05 | 2698 |
| Cl | 5 | 6.05 | 2698 |
| Cl | 7 | 6.09 | 2698 |
| Cl | 8 | 6.05 | 2698 |
| Cl | 9 | 6.05 | 2698 |
| Cl | 11 | 6.26 | 2698 |
| Cl | 12 | 5.92 | 2698 |
| Cu | 1 | 6.38 | 2792 |
| Cu | 2 | 6.55 | 2792 |
| Cu | 4 | 6.3 | 2792 |
| Cu | 5 | 6.3 | 2792 |
| Cu | 7 | 6.38 | 2792 |
| Cu | 9 | 6.51 | 2792 |
| Cu | 10 | 6.09 | 2792 |
| Cu | 11 | 6.09 | 2792 |
| Cu | 12 | 6.09 | 2792 |
| Am | A | 5.42 | 1591 |
| Am | B | 5.46 | 1591 |
| Am | C | 5.5 | 1591 |
| Am | D | 5.33 | 1591 |
| Am | E | 5.63 | 1591 |
