# Supplementary material for: Parallel altitudinal clines reveal trends in adaptive evolution of genome size in Zea mays
Source: PLoS Genet. 2018 May 10;14(5):e1007162. doi: 10.1371/journal.pgen.1007162 (PMC5944917; doi:10.1371/journal.pgen.1007162)
Supplement: S6 Table — (PDF) [file pgen.1007162.s016.pdf]

S6 Table. Repeated measures of genome size from maize inbreds lines

SampleID	Measure1	Measure2
Ki3	5.96	6.01
Ky21	5.50	5.63
NC358	5.92	5.88
B73	5.42	5.46
CML247	6.05	6.05
CML52	6.22	6.13
P39	5.50	5.50
H95	5.84	5.80
A188	5.71	5.63
K55	5.80	5.84
K64	5.71	5.80
NC33	5.75	5.80
Pa762	5.88	5.80
K4	5.54	5.50
M14	5.84	5.80
B64	5.92	5.71
T8	5.59	5.59
B84	5.63	5.50
IDS28	5.46	5.50
CH9	5.67	5.71
CML5	6.13	6.13
CML10	5.96	6.05
CML220	6.13	6.30
CML331	6.05	6.05
CML332	6.05	6.09
NC310	5.67	5.80
NC318	5.84	5.88
NC336	6.26	6.13
NC344	5.67	5.67
SA24	5.84	5.71
SA55	6.05	5.96
Mo46	5.96	5.88
CML264	6.01	5.96
DE1	5.80	5.63
RIMMA0806	5.71	5.67
