# Supplementary material for: Parallel altitudinal clines reveal trends in adaptive evolution of genome size in Zea mays
Source: PLoS Genet. 2018 May 10;14(5):e1007162. doi: 10.1371/journal.pgen.1007162 (PMC5944917; doi:10.1371/journal.pgen.1007162)
Supplement: S7 Table — (PDF) [file pgen.1007162.s017.pdf]

S7 Table. ***Mexicana*** **Population IDs and number of individuals used for FISH analyses**

Population ID	Number of individuals
RIMME0021	12
RIMME0026	12
RIMME0028	12
RIMME0029	12
RIMME0030	12
RIMME0031	12
RIMME0032	12
RIMME0033	12
RIMME0034	9
RIMME0035	12
