# Supplementary material for: Parallel altitudinal clines reveal trends in adaptive evolution of genome size in Zea mays
Source: PLoS Genet. 2018 May 10;14(5):e1007162. doi: 10.1371/journal.pgen.1007162 (PMC5944917; doi:10.1371/journal.pgen.1007162)
Supplement: S8 Table — Calculated altitudinal coefficients (β) from the models testing for altitudinal selection. β values are given in units of megabases per meter. * = p-value<0.05; ** = p-value<0.005. (PDF) [file pgen.1007162.s018.pdf]

S8 Table. Altitudinal coefficients from selection models using maize landraces and highland teosinte. Calculated altitudinal coefficients (β) from the models testing for altitudinal selection. β values are given in units of megabases per meter. *=p-value<0.05; **=p-value<0.005

β	Maize in MA	Maize in SA	Mexicana
Genome size	-0.1082932	-0.1543104	-0.2698874
Knob180	-0.004977329	-0.04344294	-0.009973119
TR1	-0.005650659	-0.00783801	-0.009217123
TE	-16797840	20189310	0.07281335
**p-value**	Maize in MA	Maize in SA	Mexicana
Genome size	0.002860943	0.00001214581	0.000153224
Knob180	0.6955793	0.02764277	0.9040046
TR1	0.02032799	0.01453535	0.005528401
TE	0.05931269	0.06856391	0.1843363
