# Supplementary material for: Parallel altitudinal clines reveal trends in adaptive evolution of genome size in Zea mays
Source: PLoS Genet. 2018 May 10;14(5):e1007162. doi: 10.1371/journal.pgen.1007162 (PMC5944917; doi:10.1371/journal.pgen.1007162)
Supplement: S9 Table — In plant ID’s, the first digit indicates the mother, while the second is a unique identifier for each individual. (PDF) [file pgen.1007162.s019.pdf]

S9 Table. Measured growth rate for Tenango del Aire population in the growth chamber experiment. In plant ID's, the first digit indicates the mother, while the second is a unique identifier for each individual.

ID	Genome Size (pg/2C)	Initial	24hr (cm)	48hr (cm)	72hr (cm)
1-1	6.11	1.7	6.6	11.3	17.6
1-11	5.98	1	3.7	7.6	12.7
1-12	5.94	3	6.8	11.1	16.2
1-14	5.86	7	11.3	16.3	20.5
1-4	6.29	5.3	9.6	18	NA
1-5	5.92	9.4	10.8	12	12.5
1-6	5.72	3.5	7	11.5	NA
1-7	5.91	5.8	8	10.2	11.9
1-8	6.14	4.9	7.7	11.3	14.6
11-2	5.92	7.6	9	12.6	17.9
11-3	5.8	2.5	6.9	11.6	16.8
11-4	5.8	6.4	11.3	17	21.2
11-6	5.84	2.9	6.1	9.6	13.5
3-2	6.26	7.7	9.4	14.8	17
3-4	6.05	7.4	12.7	16.9	20.9
3-6	6.09	2.9	3.9	12.5	17
3-7	6.22	8.9	14.8	21	26
4-1	5.75	4.5	10	15.6	22.5
4-2	5.92	7.5	11.3	13.4	18.6
4-4	6.34	4.5	8.6	13.5	18.3
4-6	6.13	5.7	8.9	10	14.6
4-7	5.96	6	11.3	17.6	24.3
8-1	5.96	6.1	9.1	13.1	16.5
8-10	6.01	4.1	7.5	11.5	12.7
8-11	5.92	3.5	7.3	13	19.4
8-12	5.84	3.5	6.8	11.2	16.4
8-13	6.05	8	13.1	17.3	21.8
8-14	5.75	3.5	7.3	10.9	15.5
8-15	6.09	2.1	6	11.3	16.6
8-16	6.13	5.1	10.3	16.5	23
8-2	6.17	6	9.5	14	17.5
8-3	6.55	4.7	9.2	12.5	18.2
8-4	6.34	4.2	7	10.6	16
8-5	6.43	3	5.1	7.9	11.2
8-7	6.01	8.6	13.6	18.9	NA
8-8a	6.51	6.3	8	NA	10.7
8-8b	6.09	2.5	4.1	7.8	12.2
8-9	6.22	8.1	12	16.8	22.1
a-1	5.92	2	5.9	9.9	14.7
a-10	6.01	3.5	4.8	7.2	10.5
a-12	5.84	3.2	6.5	10	13.2
a-2	5.71	7.5	12.9	18.5	23
a-3	5.96	14.1	16.2	19	20.1
a-4	6.09	13	18	22.6	26
a-5	5.96	5.5	9.2	14.5	19.3
a-6	5.92	5.3	7.7	12.2	16

a-7	5.8	5.4	7.2	10.5	15
a-8	6.22	4.2	8.6	14	18.9
a-9	6.05	8	11.4	15	18.1
b-1	6.01	3.3	5.5	9.5	15
b-2	6.55	2	5.6	9.6	13.9
b-3	5.8	6.2	11.3	16.8	19.2
b-4	6.3	8.3	12.5	14	17.5
b-6	5.96	9.4	12.9	18.3	24.6
b-7	5.92	2.4	5.2	12	17.4
bulk-1	6.01	8.6	12.1	15.9	20.5
bulk-10	6.43	2	5.5	10.5	16
bulk-11	6.17	5.5	9.2	14.8	18.5
bulk-12	5.71	8.4	13.3	16.2	22.1
bulk-13	5.96	5.1	8.9	12	17
bulk-14	6.13	5.3	8.1	12.7	17.3
bulk-16	5.96	1.6	4.5	8.2	14
bulk-17	6.05	2.7	6.5	11.4	15.2
bulk-18	5.92	1.2	4.5	7.5	12.5
bulk-19	6.07	4	6	10	14.6
bulk-20	6.1	3.7	6.3	11.1	14.7
bulk-21	5.98	1.8	4.8	6.9	13.7
bulk-22	6.01	6.1	9	12.8	18.1
bulk-23	5.96	0.9	6	8	13.6
bulk-24	5.98	3	4.4	8	13.6
bulk-25	5.88	8.6	13.9	16.6	22
bulk-26	5.85	3	6.9	10.9	15.1
bulk-28	5.85	2	6	10.5	15.9
bulk-29	5.85	7.7	11.8	16.2	20.5
bulk-30	6.11	5.6	8.3	13	18
bulk-31	6.01	2.5	5.5	7.9	12
bulk-32	5.92	3.5	8.4	13.7	19.2
bulk-35	6.01	6	11.4	18	23.6
bulk-36	5.96	3.9	10.1	16.6	24.2
bulk-37	6.01	2.6	5.5	9.2	16.8
bulk-38	5.9	0.5	4.6	10	16.8
bulk-4	6.05	6.4	9.7	16	19
bulk-40	6.1	6.7	12.2	19.1	25
bulk-42	6.13	5.5	10.1	15.2	20.4
bulk-5	5.96	1.6	4.8	9.8	14.5
bulk-6	6.13	6	9	13.5	16.6
bulk-7	6.17	3.5	6.8	13	16.7
bulk-8	6.43	5.2	8.4	12.6	17
bulk-9	6.05	5.4	8.4	13.1	17.6
c-1	5.71	6.4	8.7	14.3	20
c-10	5.88	1.9	6.6	12	18.6
c-11	5.84	2.8	6.1	11.1	15.7
c-2	5.59	4.4	7.3	12	17.5
c-3	5.67	5	11	17.1	24.3
c-4	5.63	3.5	5	9	12.8
c-5	5.71	5.5	8.8	13.5	17.6
c-6	5.84	5.5	10.3	16.5	22.3
c-7	5.71	NA	22.8	28.8	34.3

c-8	5.84	11.5	18	24.1	30.5
c-9	5.63	6	10.6	16.1	22.1
d-1	5.96	6.2	11.5	16.6	22
d-10	6.09	1.9	6.4	11.8	18
d-12	6.05	2.1	6	11.6	17.8
d-13	6.13	1.6	6	11	17.4
d-14	6.13	3.2	7.2	13.1	20.3
d-15	5.84	2.6	7.8	13	NA
d-16	6.01	4.6	9.8	15.6	22
d-17	5.75	2.1	6	10.5	14.6
d-18	5.84	8	13.4	19.5	26.5
d-2	5.84	NA	20.5	27.8	34.5
d-20	6.05	3.6	9.7	16.6	24
d-3	5.88	7	12.1	18	23.5
d-4	5.84	1.7	5.3	12.3	14.2
d-6	5.75	3.5	9.4	13.2	19.3
d-7	5.84	9.5	15	18.2	22
d-8	5.75	3.1	6.8	10.1	14.7
d-9	6.09	7.5	10.1	14.8	18
e-1	5.84	1.7	2	8.5	13.4
e-10	6.26	3.5	7.8	13.1	19
e-11	5.88	3.5	8.6	15.5	22.3
e-12	5.92	4.3	9.5	16.6	24.1
e-13	5.63	5	10.5	18	25
e-14	5.96	2.7	7.3	13.5	19.9
e-15	5.8	2.3	7	11.6	17.2
e-16	6.3	5.6	10	15.8	21.6
e-17	6.01	9.1	14.3	19.2	23.5
e-18	5.84	1.5	4.5	8.4	12.5
e-19	5.92	5.4	9.5	14.5	18.6
e-3	5.96	5	11.9	12.2	15.6
e-4	5.92	1.5	4.3	8.8	14.7
e-6	6.05	3.3	6.4	11.5	14.1
e-8	6.01	4.5	8	13.2	21.2
e-9	5.92	2.5	6	13.8	21.5
f-1	5.88	7.5	11	16.2	22.2
f-2	5.42	6.2	8.3	12.2	16.5
f-3	5.8	3.3	6.8	11.2	16.2
f-4	5.59	1.1	4	8.2	12.8
f-6	5.88	2.6	4.1	9.1	11.5
f-7	5.63	1.2	5.1	10	16
f-8	5.84	0.3	2.6	6.8	12.2
g-1	5.54	8.5	13.5	19.6	25.2
g-10	5.75	3.6	7.3	12.6	18.6
g-2	5.71	8.5	13.4	18.5	22.6
g-3	5.75	5.3	8.8	13.4	17.4
g-4	5.84	8	11.6	15.2	21
g-5	5.67	3.2	6.2	9.6	15.5
g-6	5.71	3.5	7.3	12.7	18.6
g-7	6.01	4.6	8	11.4	16
g-8	6.05	3.5	6.9	11.5	17
g-9	5.84	10.1	15.3	21.3	30.5

h-10	5.96	4.6	8	13.1	17
h-13	6.09	3.5	8.5	13.4	18.3
h-2	5.96	4	8	12.3	16
h-4	6.01	3	5.6	8.6	12.5
h-5	5.84	2.4	5.3	10	15.5
h-6	6.13	3	7	11.5	16.2
h-7	6.09	7	11.5	16	20.3
h-8	6.43	8.4	13.5	21	27.1
h-9	5.92	1.8	5.3	10.8	17.1
i-1	5.88	4.5	7.2	12.2	17
i-10	5.96	5	8.4	12.6	18.3
i-12	5.75	3.1	6.4	9.6	12.2
i-13	5.88	3.6	7.3	11.6	16.6
i-2	6.01	6	10.5	15.5	20.6
i-3	5.84	7.5	10.1	14.5	19.5
i-4	5.88	5.2	14.5	20.3	26
i-7	5.8	3.5	6.9	12.1	14.5
i-9	5.71	4.3	8.9	13.1	17.6
j-1	6.09	2.1	3.8	9.4	12
j-10	6.13	3.5	8.5	13.5	19.4
j-11	5.88	0.9	5	11	NA
j-2	6.38	7.4	10.5	14.8	18.3
j-3	6.3	2	6	10.5	14.6
j-4	5.88	3.5	8.2	11.7	16.7
j-5	5.96	4.7	8.4	12.6	17.3
j-7	5.92	1.5	5.5	9	11.7
j-8	6.34	2.4	5.6	10.5	15.6
j-9	6.09	3.5	7.8	13	18.5
k-10	5.74	1.5	6	11.5	18.2
k-11	5.5	2.5	6.5	11.8	17.9
k-14	5.94	2.1	5.6	9.8	14.1
k-15	5.77	1	4.5	9	13.6
k-16	5.82	1.1	4	8.1	12.5
k-17	5.93	5.6	10.4	15.9	21.5
k-3	5.88	1.4	3	8.7	13.5
k-5	5.74	4.8	10.5	18	30.1
k-6	5.9	2.1	6.1	11.6	18.1
k-7	5.66	7.7	13.3	19.5	25.1
k-8	6.01	4.2	9.6	16.6	23.6
k-9	5.77	3.4	6	9.8	11.5
l-10	5.75	7.4	11	16	21.5
l-11	5.8	10.6	15.7	21.2	25.5
l-12	5.71	4.6	9	14	19.5
l-13	6.05	7.4	7.9	16.9	22.4
l-15	6.22	0.3	4.5	9.9	15.5
l-16	5.92	13.2	18.5	23.6	27.2
l-3	5.88	4.5	8.9	14.1	18.6
l-6	5.96	6.9	11.5	16.5	21.1
l-7	6.01	8	14.3	22.9	29
l-8	6.17	3.4	6.7	11.6	14.5
l-9	5.96	4.2	8.9	15	21
